# Contributions of γ-Aminobutyric Acid (GABA) Receptors for the Activities of *Pectis brevipedunculata* Essential Oil against *Drosophila suzukii* and Pollinator Bees

**DOI:** 10.3390/plants13101392

**Published:** 2024-05-17

**Authors:** Sabrina Helena da Cruz Araujo, Javier Guillermo Mantilla-Afanador, Thiago Svacina, Tarciza Fernandes Nascimento, Aldilene da Silva Lima, Marcos Bispo Pinheiro Camara, Luis Oswaldo Viteri Jumbo, Gil Rodrigues dos Santos, Cláudia Quintino da Rocha, Eugênio Eduardo de Oliveira

**Affiliations:** 1 Departamento de Entomologia, Universidade Federal de Viçosa, Viçosa 36570-900, MG, Brazil; sabrinahelena@alumni.usp.br (S.H.d.C.A.);; 2 Grupo de Pesquisa em Microbiologia e Biotecnologia Agroindustrial, Universidad Católica de Manizales, Rua 23 N. 60-63, Manizales 170001, Colombia; jmantilla@ucm.edu.co; 3 Programa de Pós-Graduação em Biotecnologia, Universidade do Federal do Tocantins, Gurupi 77402-970, TO, Brazilluisviterijumbo3737@gmail.com (L.O.V.J.); gilrsan@uft.edu.br (G.R.d.S.); 4 Departamento de Química, Universidade Federal do Maranhão, São Luís 65080-805, MA, Brazil; aldileney@hotmail.com (A.d.S.L.); rocha.claudia@ufma.br (C.Q.d.R.); 5 Programa de Pós-Graduação em Ciências Florestais e Ambientais, Universidade Federal do Tocantins, Gurupi 77402-970, TO, Brazil; 6 Programa de Pós-Graduação em Biologia Animal, Universidade Federal de Viçosa, Viçosa 36570-900, MG, Brazil

**Keywords:** biorational pesticides, plant-based insecticides, non-target organisms, molecular docking predictions

## Abstract

The γ-aminobutyric acid (GABA) receptors play pivotal roles in the transmission of neuronal information in the nervous system of insects, which has led these proteins to be targeted by synthetic and natural products. Here, we assessed the insecticidal potential of the essential oil of *Pectis brevipedunculata* (Gardner) Sch. Bip., a neotropical Asteraceae plant used in traditional medicine, for controlling *Drosophila suzukii* (Matsumura) adults by feeding exposure. By using in silico approaches, we disentangle the contribution of GABA receptors and other potential neuronal targets (e.g., acetylcholinesterase, glutathione-S-transferases) in insects that may explain the essential oil differential activities against *D. suzukii* and two essential pollinator bees (*Apis mellifera* Linnaeus and *Partamona helleri* Friese). Neral (26.7%) and geranial (33.9%) were the main essential oil components which killed *D. suzukii* with an estimated median lethal concentration (LC_50_) of 2.25 µL/mL. Both pollinator forager bee species, which would likely contact this compound in the field, were more tolerant to the essential oil and did not have their diet consumptions affected by the essential oil. Based on the molecular predictions for the three potential targets and the essential oil main components, a higher affinity of interaction with the GABA receptors of *D. suzukii* (geranial −6.2 kcal/mol; neral −5.8 kcal/mol) in relation to *A. mellifera* (geranial −5.2 kcal/mol; neral −4.9 kcal/mol) would contribute to explaining the difference in toxicities observed in the bioassays. Collectively, our findings indicated the involvement of GABA receptors in the potential of *P. brevipedunculata* essential oil as an alternative tool for controlling *D. suzukii*.

## 1. Introduction

Plants of the botanical family Asteraceae comprise most of the angiosperms and are considered one of the largest among the phanerogams [1]. Many of these species are highly valued for cultivation due to their biological value, especially for essential oil extraction. These oils may contain bioactive compounds with a wide range of properties, from antibacterial to insecticidal, larvicidal, acaricide, nematicidal, and antifungal action [2]. Plants belonging to this family can be found in several regions, including tropical, subtropical, and temperate, growing in the most varied habitats [3].

Among the diversity of plants from the Asteraceae family, there are those belonging to the genus Pectis, which comprises about 76 neotropical species. One of such species is *Pectis brevipedunculata* (Gardner) Sch. Bip. It is a small plant, rich in secondary metabolites, and native to dry and rocky environments [4,5]. The essential oil of *P. brevipedunculata* is mainly composed of citral, α-pinene, and limonene [4,6], which have antimicrobial, antioxidant, anti-inflammatory, and antidepressant potential [7,8,9,10]. It was also recently demonstrated that the essential oil of this species has an acaricidal effect and is selective for ladybugs and lacewings [5], raising interest in this species as a potential source for the development of novel biorational pesticides.

Given the diversity of the beneficial compounds found in the essential oil of *P. brevipedunculata*, the focus of this work is directed to an issue of economic and agricultural importance. The fly *Drosophila suzukii* (Matsumura) (Diptera: Drosophilidae) is originally from Asia, being described for the first time in Japan in 1931 [11]. It is also known as spotted-wing drosophila, had its presence registered in Brazil in 2013 [12], and since then it has been responsible for causing serious damage to several soft summer and thin-skinned fruit crops [13].

This fly represents a pest of great economic importance due to its impact on several soft-skinned fruit crops, such as strawberries, grapes, raspberries, and blackberries [12]. Unlike other flies, *D. suzukii* has a distinctive feature in its females: a serrated ovipositor, which allows them to deposit their eggs inside healthy ripening fruits. This oviposition results in openings in the fruit, which, in addition to facilitating contamination by microorganisms, creates an environment conducive to larval feeding and development, leaving the fruit softened and accelerating decomposition. This larval activity causes significant damage leading to a considerable productivity loss and the reduction in the market value of the affected crops [11,14].

Until now, the search for an effective and environmentally friendly approach against *D. suzukii* has been challenging due to the difficulty of identifying an infestation by this species, since the females lay their eggs inside the fruits [15]. Pest management alternatives for this fly have focused mainly on the use of chemicals, which can generate resistance and be harmful to beneficial insects, such as pollinators [16]. Honey bees and stingless bees, for instance, are essential to high-yield strawberry fields, enhancing fruit weight and value by complementary pollination [17,18]. Pollinator-safe approaches are needed to be urgently applied in integrated pest management programs, even more so considering that the majority of strawberry landscapes are formed by everbearing genotypes (constant flowering, fruit ripening, and harvesting) [19,20]. In this context, a control approach is sought that is based on selective natural products which are rapidly degraded and do not leave residues in different plant parts, causing less damage to the environment and not affecting the beneficial organism’s activities [13,21,22].

Thus, we sought to evaluate the action of the essential oil of *P. brevipedunculata* against *D. suzukii* in addition to evaluating its selectivity for *Apis mellifera* (Linnaeus) and *Partamona helleri* (Friese) bees. Furthermore, based on the major compounds neral (=β-citral) and geranial (=α-citral) derived from the essential oil of *P. brevipedunculata*, we are predicting its potential mode of action and selectivity towards the non-target organisms. Our predictions analyzed the binding interactions between neral and geranial and the γ-aminobutyric acid (GABA) receptors in *D. suzukii* and *A. mellifera* via molecular docking, identifying the GABA receptor as a possible cellular target. Therefore, this is the first report of insecticidal activity of *P. brevipedunculata* essential oil, demonstrating its selectivity towards pollinators, and correlating its bioactivity to potential physiological targets (e.g., GABA receptors).

## 2. Results

### 2.1. Essential Oil Extraction and Composition

The chemical composition of *P. brevipedunculata* essential oil is described in Table 1. Thirty-four compounds were identified, and the essential oil consists mainly of oxygenated monoterpenes. The main components were neral (=β-Citral) corresponding to 26.7% and geranial (=α-Citral) corresponding to 33.9% of the total composition. The yield of essential oil extraction was 1.8%.

### 2.2. Toxicity to D. suzukii

**Figure 1:**
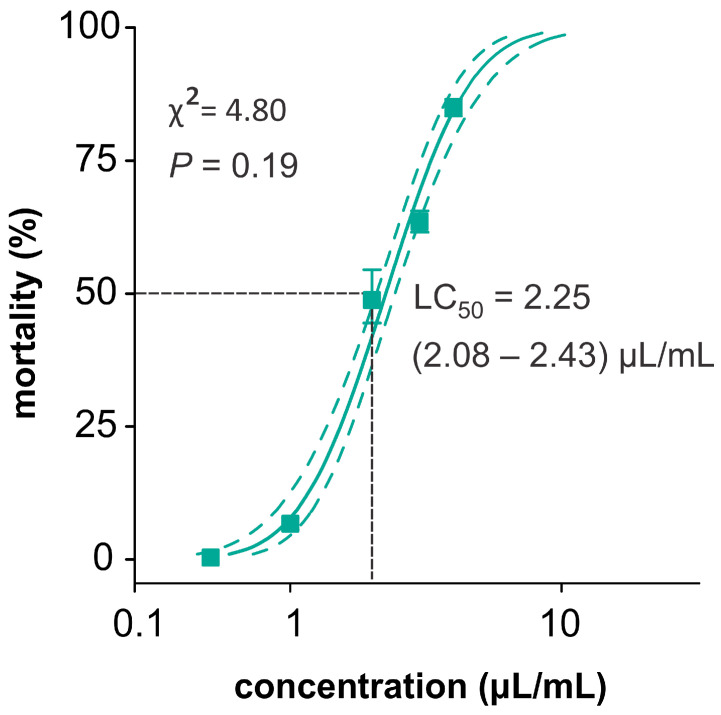
*Pectis brevipedunculata* essential oil affecting the survival of *Drosophila suzukii*. Mortality was assessed after 24 h of exposure by feeding. The LC_50_ represents the lethal concentration average followed by their upper and lower limits. The bars indicate the standard errors. χ^2^ = Chi-square test; *p* = Probability value.

Exposure to essential oil by feeding caused mortality in *D. suzukii* (Figure 1). The lethal concentration that killed 50% of the insects (LC_50_) estimated for the mortality-concentration curve (χ^2^ = 4.80, *p* = 0.19) was 2.25 (2.08–2.43) µL/mL.

### 2.3. Essential Oil Selectivity to Bees

**Figure 2:**
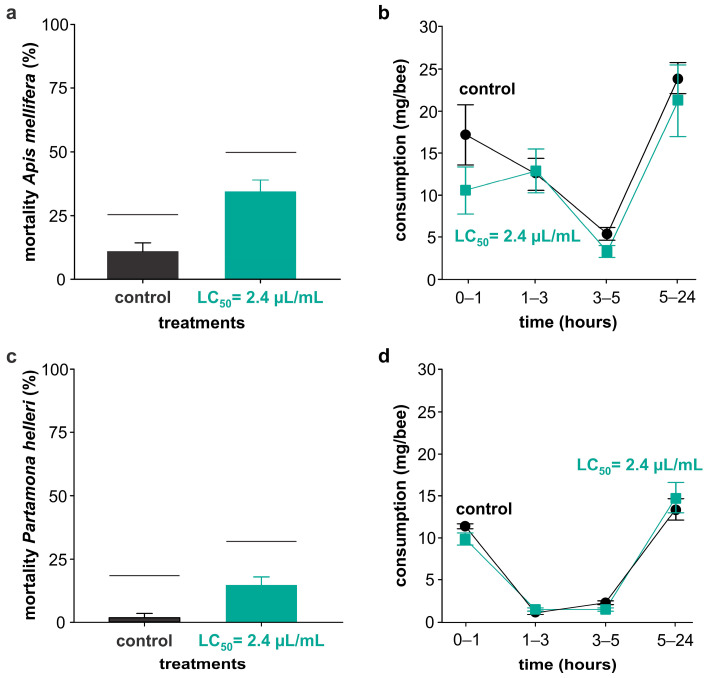
Effects of exposure to *Pectis brevipedunculata* essential oil on the bees of species *Apis mellifera* and *Partamona helleri*. The bees were exposed to the essential oil by feeding for 5 h. After this period, the insects received a diet containing only sugar syrup (50% [*m/v*]). After 24 h, mortality was evaluated for *A. mellifera* (**a**) and *P. helleri* (**c**). During the entire period, the consumption of diets for *A. mellifera* (**b**) and *P. helleri* (**d**) was determined by weighing the diets. The control represents the insects that received diets containing dimethylsulfoxide (1.2 µL/mL), polysorbate 20 (0.6 µL/mL), and sugar syrup. LC_50_ represents the lethal concentration obtained from the concentration-mortality curve for *Drosophila suzukii*. The vertical bars indicate the standard errors. The horizontal bars indicate the significant difference according to the *t*-test (*p* < 0.05).

**Figure 3:**
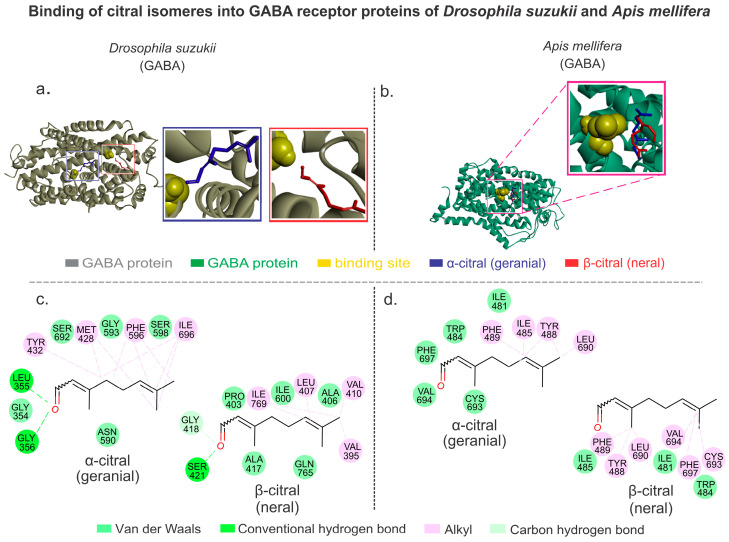
Citral isomers interacting with GABA receptors from *Drosophila suzukii* and *Apis mellifera*. (**a**) The protein 3D structure of the *D. suzukii* GABA receptor with the citral isomers. (**b**) The protein 3D structure of the *A. mellifera* GABA receptor with the citral isomers. (**c**) The 2D interaction maps showing the molecular interactions between amino acids from the binding site environments of *D. suzukii* GABA receptor with the citral isomers. (**d**) The 2D interaction maps showing the molecular interactions between amino acids from the binding site environments of *A. mellifera* GABA receptor with the citral isomers.

Exposure to the essential oil LC_50_ (i.e., 2.4 µL/mL) by feeding killed more *A. mellifera* (Figure 2a) and *P. helleri* (Figure 2c) bees compared to the control. For both species of bees, there was a reduction in the consumption of both diets up to the fifth hour, with an increase in consumption after this period (Figure 2b,d). Despite that, the repeated measures ANOVA (*F* = 3.86; *p* = 0.067) revealed that there was no effect of the essential oil on diet consumption (Table 2). Likewise, the interactions between essential oil and bee species (*F* = 3.27; *p* = 0.089), between essential oil and time (*F* = 0.76; *p* = 0.53), and between essential oil, bee species, and time (*F* = 0.57; *p* = 0.64) had no significant effect on diet consumption. However, there was the expected influence of species (*F* = 76.6; *p* < 0.0001), time (*F* = 380.2; *p* < 0.0001), and the interaction between these two factors (*F* = 61.0; *p* < 0.0001) on diet consumption. These results are consistent with the consumption shown in Figure 3b,d.

### 2.4. Interactions between Citral Isomers GABA Receptors of D. suzukii and A. mellifera

Based on our results of the insecticidal effect against *D. suzukii* of *P. brevipedunculata* essential oil, we hypothesized that principal oil components may interact with three possible targets of *D. suzukii*: GABA receptors, acetylcholinesterase (AChE), and glutathione-S-transferase (GST) enzymes. By comparing the differences in affinity between interactions for *D. suzukii* and *A. mellifera* targets, we sought to explain the differences in essential oil toxicity between *D. suzukii* and pollinator bees. The quality parameters, estimated according to the Procheck tool, indicated that the calculated 3D structures were adequate for the GABA receptor of *D. suzukii* and *A. mellifera*. Thus, the constructed GABA receptor protein model of *D. suzukii* and *A. mellifera* highlighted the values of Ramachandran favored with 92.2% and 90%. The z-score of the PROSA server indicated that the GABA receptor models *D. suzukii* and *A. mellifera* have both global and local scores in the acceptance zone and have energy stability. The global analysis of the model GABA receptor with PROSA showed a z score of 4.47 for *D. suzukii* and 4.52 for *A. mellifera*, indicating no significant deviation of the predicted structures from the native proteins of similar size. The selected templates for the modeling of AChE by homology highlighted the values of Ramachandran favored with 90.4% and a QMEAN factor of 0.97 corresponding to *D. suzukii*, and 90.1% and 0.82 corresponding to *A. mellifera*. On the other hand, the template for GST highlighted the values of Ramachandran favored with 96.5% and a QMEAN factor of 0.3 corresponding to *D. suzukii*, and 97.1% and 0.1 corresponding to *A. mellifera*. The Ramachandran plots are available in Figure S1.

The GABA receptor of *D. suzukii* exhibited higher energy affinities (AutoDockVina affinity energy) when complexed with α-citral (geranial) molecule (−6.2 kcal/mol) compared to *A. mellifera* (−5.2 kcal/mol). Likewise, *D. suzukii* exhibited higher energy affinities with β-citral (neral) (−5.8 kcal/mol) compared to *A. mellifera* (−4.9 kcal/mol). It was characteristic that the two biomolecules showed different binding pockets in *D. suzukii* (Figure 3a,b). The α-citral (geranial) complex with *D. suzukii*-related GABA receptor showed van der Waals interactions with GLY354, SER692, GLY593, SER598, and ASN590; hydrogen bond interactions with LEU355 and GLY356; and alkyl interactions with TYR432, MET428, PHE596, and ILE696. Conversely, the interactions of *A. mellifera* GABA receptor protein showed a different profile, exhibiting van der Waals bond interactions with CYS693, VAL694, PHE697, TRP484, and ILE481; and alkyl interactions with PHE489, ILE485, TYR488, and LEU690 (Figure 3c). The complex formed between β-citral (neral) and *D. suzukii*-related GABA receptor protein showed hydrogen bond interactions with SER421; van der Waals interactions with PRO403, ALA417, ILE600, and GLN765; alkyl interactions with ILE769, LEU407, and VAL395; and carbon–hydrogen bond interactions with GLY418, whereas the complex formed between β-citral (neral) and *A. mellifera* showed van der Waals interactions with ILE485, ILE481, and TRP484; and alkyl interactions with PHE489, TYR488, LEU690, VAL694, PHE697, and CYS693 (Figure 3d). The absence of hydrogen interactions in *A. mellifera* of both citral isomers is consistent with the lower affinity energy and therefore the lower stabilities of the citral molecules compared to *D. suzukii* where hydrogen interactions is present.

The GABA receptor from *D. suzukii* presented differences in affinity energy (AutoDockVina affinity energy kcal/mol) with fipronil (−9.1 kcal/mol) compared to *A. mellifera* (−7.3 kcal/mol). The fipronil complex with the GABA receptors of *D. suzuki* showed van der Waals bond interactions with SER425, GLY593, PHE602, SER591, LEU592, LEU688, LEU355, GLY356, TYR432, ASN590, MET428, and VAL700; carbon–hydrogen bond interactions with SER598, SER692, GLY354, and ILE696; halogen interactions with ALA695, TYR351, VAL353, and PHE596; and alkyl interactions with VAL699. On the other hand, the fipronil complex with *A. mellifera* GABA receptors showed van der Waals bond interactions with VAL775, PHE778, ASP782, THR787, TYR783, PRO786, TYR718, SER721, ILE722, SER779, and MET725; hydrogen bond interactions with ASN556, HIS717, and GLU784; and alkyl interactions with ALA552 (Figure S2).

The *D. suzukii*-related AChE did not present significant differences in energy affinity (AutoDockVina affinity energy kcal/mol) with α-citral (geranial) (−6.3 kcal/mol) compared to *A. mellifera* (−6.1 kcal/mol). Likewise, *D. suzukii* did not exhibit differences in energy affinity with β-citral (neral) (−5.9 kcal/mol) compared to *A. mellifera* (−6.3 kcal/mol). The α-citral (geranial) complex with *D. suzukii* -related AChE showed van der Waals bond interactions with GLY188, TYR110, TYR361, TRP358, ASP412, and TYR411 and alkyl interactions with PHE408, PHE367, and Pi-Sigma interactions with TYR407 (Figure S3). The α-citral (geranial) complex with *A. mellifera* showed hydrogen bond interactions with SER171; van der Waals bond interactions with GLY167, PHE386, and ASP390; Pi-Sigma interactions with TYR339; and alkyl interactions with TRP336, TYR389, TYR104; and PHE345. On the other hand, the complex formed between β-citral (neral) and *D. suzukii*-related AChE showed van der Waals bond interactions with HIS517, GLY187, and LEU516; alkyl interactions with PHE367, PHE408, TRP120, and TYR108; and Pi-sigma interactions with TYR407. On the other hand, the β-citral (neral) complex with *A. mellifera* showed hydrogen bond interactions with LEU343; van der Waals bond interactions with ILE342, ASP390, TRP336, TYR389, and TYR106; and alkyl interactions with TYR104, TYR385, TYR339, and PHE345.

The *D. suzukii*-related GST presented high affinity energy (AutoDockVina affinity energy kcal/mol) with α-citral (geranial) (−4.7 kcal/mol) compared to *A. mellifera* (−5.1 kcal/mol). Likewise, *D. suzukii* did not exhibit differences in energy affinity with β-citral (neral) (−4.3 kcal/mol) compared to *A. mellifera* (−5.3 kcal/mol). The α-citral (geranial) complex with *D. suzukii* -related GST showed hydrogen bond interactions with GLN133; van der Waals bond interactions with ASN186, THR182, HIS108, THR140, VAL137, ILE136, ALA191, and LEU192; alkyl interactions with MET94, LEU179, and LYS178; and carbon–hydrogen bond interactions with LEU190 (Figure S4). Conversely, the α-citral (geranial) complex with *A. mellifera* showed hydrogen bond interactions with ASN10; van der Waals bond interactions with VAL11, ILE34, HIS36, PHE9, and ARG110; alkyl interactions with TYR109 and PHE209; and carbon–hydrogen bond interactions with THR35. On the other hand, the complex formed between β-citral (neral) and *D. suzukii* related GST showed van der Waals bond interactions with VAL150, LYS147, GLU154, VAL151, VAL171, THR166, and LEU167; and alkyl interactions with LYS163. Conversely, the β-citral (neral) complex with *A. mellifera* showed hydrogen bond interactions with ARG110; van der Waals bond interactions with HIS36; and alkyl interactions with VAL11, PHE9, TYR109, and PHE206.

## 3. Discussion

Here, we demonstrated that the *P. brevipedunculata* essential oil showed a composition rich (>60%,) in citral (neral + geranial), which is in accordance with previous investigations [4,5,7]. As demonstrated by other plant essential oils, the composition of *P. brevipedunculata* essential oil exhibits seasonal variations [5], but always with neral and geranial appearing among the major components, with contents varying from about 50% to over 80% of the *P. brevipedunculata* essential oil [4,5,7,8]. Thus, it is reasonable to expect that this essential oil’s efficacy towards *D. suzukii* may rely on the actions of the major compounds, although a joint action of several components cannot be ruled out.

Several studies have elucidated the biological activities of essential oils containing geranial, neral, or both (collectively known as citral), demonstrating their efficacy against *D. suzukii* [23,24,25]. For instance, the essential oil of *Melaleuca teretifolia* Endl. (72.7% citral), as well as the compounds neral and geranial, showed efficient toxicity, by contact and fumigation, against the males and females of *D. suzukii* (LC_50_ = 2.36–2.97 mg/L) [23]. In the same study, higher contact toxicity was reported (LC_50_ = 2.27–4.23 µg/insect). Similar results were observed for *Leptospermum citratum* (Challinor, Cheel and Penfold) (51.2% citral) essential oil [24]. The essential oil of *Litsea cubeba* (Lour.) Pers. (63.5% citral) and citral applied alone presented repellent and fumigant actions in addition to contact larvae toxicity, which was related to histological damage and changes in protein content [25]. Additionally, several essential oils rich in geranial and neral caused toxicity, strong repellency, and reduced oviposition to *D. suzukii*, and are associated with low toxicity for the endoparasitoid *Trichopria anastrephae* (Lima) [26].

Another possibility for the essential oil’s effectiveness may be related to the repellent action of citral. Since exposure to essential oil was performed by feeding, a repellent action may result in the lower consumption of diets, and thus, the condition of starvation contributes to increased mortality. The repellent action of citral for *D. suzukii* has already been demonstrated through choice experiments in which most females preferred to migrate to containers with a mixture of vinegar and wine without the presence of citral [27]. Repellent actions attributed to citral alone or other citral-rich essential oils have also been demonstrated for mosquitoes such as *Aedes aegypti* (Linnaeus *in* Hasselquist) ([28], *Aedes albopictus* (Skuse) [29,30] and *Culex pipiens* (Linnaeus) [29]; and for flies such as melon fly, *Bactrocera cucurbitae* (Coquillett) [31] and stable fly, *Stomoxys calcitrans* (Linnaeus) [32]. In addition to the repellent action, the reduction in diet consumption can be related to a deterrent effect caused by citral, as already demonstrated for aphids especially *Myzus persicae* (Sulzer) [33].

Here, we also investigated the impact of *P. brevipedunculata* essential oil on the honeybee species *A. mellifera* and the stingless bee *P. helleri*. The use of plant-based pesticides has raised concerns about their potential toxic effects on pollinators [34]. Therefore, it is crucial to rigorously assess the lethal (i.e., mortality) and sublethal (i.e., consumption alteration) effects of these biopesticides on non-target organisms to ensure the safety of bees and other beneficial insects [35]. *P. brevipedunculata* essential oil has already shown safety for other non-target organisms such as ladybugs and lacewings [5]. In this context, we aimed to evaluate the safety of using *P. brevipedunculata* essential oil as a tool to control the spotted-wing *D. suzukii* by measuring its effects on *A. mellifera* and *P. helleri* using the LC_50_ for the target. Our first finding revealed that the mortality rate of both non-target organisms was significantly lower compared to *D. suzukii*, indicating a promising outcome in terms of safety when compared to common synthetic pesticides known to have detrimental effects on bees [36,37,38].

Although *A. mellifera* and *P. helleri* exhibited less tolerance to the LC_50_ compared to control conditions, it is a noteworthy result, given the potential harm caused by synthetic pesticides. Additionally, *P. helleri* showed a lower mortality rate when exposed to the essential oil alone, consistent with previous findings [34,39]. However, it is important to note that the combination of *P. brevipedunculata* essential oil with other chemicals could lead to different outcomes. Therefore, subsequently, we examined the potential effects of the essential oil on the bees’ ability to consume syrup. Our results demonstrated that the insecticide alone did not significantly alter the feeding capacity of both bee species (*p*-value = 0.067). The analysis also revealed significant effects of time (*p*-value < 0.0001) and species (*p* < 0.0001) individually, as well as an interaction between time and species (*p* < 0.0001). These results further support the reliability of our risk-assessment procedure, indicating minimal lethal and sublethal effects.

Finally, we used in silico predictions to analyze potential modes of action of neral and geranial on *D. suzukii* and their selectivity in favor of the pollinators. Our focus relied on analyzing the binding interactions for these compounds and the physiological targets (i.e., the enzymes GST and AChE, and GABA receptors) of insecticides. GST plays a role in detoxification, catalyzing the conjugation of glutathione with toxic components, being related to insecticide resistance [40]. AChE represents the main enzymes present in the neuronal and neuromuscular junctions of insects and are the targets of many essential oils [41]. Previous studies [23] reported the efficacy of neral- and geranial-rich essential oils in controlling *D. suzukii*, demonstrating a low inhibition of AChE activity and a high inhibition of GST activity in insects treated with these compounds. Similarly, it was demonstrated that one of the mechanisms of the action of *L. cubeba* essential oil against *D. suzukii* larvae occurs through neurotoxicity by the reduction in AChE content, and this essential oil presented citral as the main component [25]. However, our analysis demonstrated that interactions with the AChE and GST enzymes are not able to explain the differences in toxicity of the essential oil to *D. suzukii* and the bees. On the other hand, the differences in the interaction affinity of neral and geranial with the GABA receptor from *D. suzukii* and *A. mellifera* suggest that this receptor is responsible for the selectivity of the essential oil. Furthermore, our predictions for the interactions of GABA receptors and the already marketable insecticide fipronil, a very well-known disruptor of GABA receptor function in insects, reinforce our findings for neral and geranial. Fipronil exhibited a higher binding affinity for the GABA receptors of *D. suzukii* (−9.1 kcal/mol) when compared to those predicted bindings in *A. mellifera* GABA receptors (−7.3 kcal/mol). However, it is worth highlighting that fipronil’s binding affinities were higher than the binding affinities of geranial and neral in both insect GABA receptors tested, which may reflect a higher efficacy to kill both insect species (i.e., *D. suzukii* and *A. mellifera*). Indeed, several studies demonstrate the toxic effects of fipronil on honey bees and stingless bees [42,43,44], including *P. helleri* [45] even at concentrations much lower than those used in our study. These undesired effects of fipronil in pollinator bees may help to explain the lack of information regarding the fipronil toxicities against *D. suzukii*, as these spotted-wing flies are severe pests in crops that are heavily dependent on bee-mediated pollination. Mutations in the GABA receptor of *D. simulans* (Sturtevant), a species phylogenetically closely related to *D. suzukii*, demonstrated that *D. simulans* GABA receptor plays a fundamental role in fipronil resistance [46].

The insect GABA receptors are the targets of various insecticides since GABA is the main inhibitory neurotransmitter in invertebrates [47]. Despite this, few research on the insecticidal action of essential oils describes the action on GABA receptors, and the description of inhibitory mechanisms are described only for some monoterpenoids and terpenes [41,48]. It is interesting to mention that due to the protective function of terpenes and terpenoids in plants, herbivorous insects have evolved to develop mechanisms of resistance to these compounds through modifications in specific sites of GABA receptors [49]. Thus, it is interesting to obtain natural products presenting other chemical groups that are effective for these receptors.

Here, the prediction of the interaction of the two citral isomers with the GABA receptors of *D. suzukii* and *A. mellifera* was satisfactory in explaining the difference in toxicity of *P. brevipedunculata* essential oil for *D. suzukii* and bees. An analysis of the interaction energies demonstrated the greater affinity of the ligands for the target proteins of *D. suzukii*, and an evaluation of the nature of the interactions demonstrated the existence of hydrogen bonds present only in interactions with the GABA receptor of *D. suzukii*. Interestingly, the binding sites of the citral isomers differ from the preferred sites described for a wide range of agonists that bind to the RDL (resistance to dieldrin) GABA subunit of *D. melanogaster* (Meigen). The main sites of interaction for these agonists were the residues of TYR109, ARG111, GLU204, PHE206, and TYR254 [50], which are in very different portions in relation to the ones we describe here. Thus, the citral isomers may perform an allosteric regulation of the GABA receptor. For *D. suzukii*, the existence of two allosteric sites may imply greater conformational changes, resulting in stronger inhibition.

## 4. Material and Methods

### 4.1. Essential Oil Extraction and Chemical Characterization

The plants of *P. brevipedunculata* were collected at the campus of Universidade Federal do Maranhão, São Luís, MA, Brazil, coordinates: 2°33′20.5″ S 44°18′32.7″ W. A sample was deposited in the Herbarium Rosa Mochel (SLUI), Universidade Estadual do Maranhão, São Luís, MA, Brazil, under No. 5287. Sampling was carried out following the Brazilian law for biodiversity protection (SisGen No. AAFB38B). The essential oil was extracted from the whole plant, excluding the roots, according to the methodology previously described [5]. For this, 100 g of the sample was air-dried for 24 h. Subsequently, the essential oil was extracted through the hydrodistillation process in a Clevenger-type apparatus for 2 h. The complete drying of the essential oil was performed using anhydrous sodium sulfate (ISOFAR, Rio de Janeiro, RJ, Brazil). The yield of the extraction was calculated in percentage *m/v* (mL per 100 g of plant sample).

The essential oil composition was determined by gas chromatography coupled with mass spectrometry (GC-MS)-QP2010 (Shimadzu Corporation, Tokyo, Japan) equipped with the GCMS Solution software version 4.3 containing libraries [51]. The capillary column used was the DB-5ms (30 m × 0.25 mm × 0.25 μm film thickness, J&W Scientific, Santa Clara, CA, USA). The following conditions were used: injector temperature of 250 °C; oven temperature programming of 35 °C for 6 min and then with a heating ramp of 10 °C min^−1^ to 240 °C remaining for 10 min; split mode injection for 1.0 µL of the sample (oil 6.0 µL: n-hexane 500 µL), split ratio 1/30; ionization by electronic impact at 70 eV; and ionization source and transfer line temperatures of 250 and 200 °C, respectively. The mass spectra were acquired through automated scanning at intervals of 0.3 s, encompassing mass fragments within the 35–400 *m*/*z* range. Quantitative information regarding the volatile components was obtained by normalizing peak areas using gas chromatography coupled with flame-ionization detection (GC-FID) from the 2010 series. This instrument was operated under conditions similar to the GC-MS. The identification of compounds was accomplished by comparing the retention indices, which were determined using a series of n-alkanes (C_8_–C_32_) obtained from Sigma-Aldrich (St. Louis, MO, USA) [52]. The identification of oil components was performed by comparing their retention indices and mass spectra (molecular mass and fragmentation pattern) with those present in the GCMS Solution system libraries [53].

### 4.2. Essential Oil Toxicity to D. suzukii

The lethal concentrations of the essential oil for *D. suzukii* flies were assessed using ingestion exposure. For this, adult insects of up to three days of emergence were used. These insects came from a laboratory *D. suzukii* population and did not have previous contact with insecticides. The flies were maintained in the laboratory at 25 ± 2 °C, 70 ± 5% relative humidity, and a 12 h photophase, and were fed an artificial diet [54]. The bioassay methodology was adapted from [55]. The essential oil was dissolved in dimethylsulfoxide—DMSO (10 µL/mL) and polysorbate 20–Tween 20 (5 µL/mL). The solutions were prepared in a sugar syrup [(20% [*m/v*]). Each 250 mL vial received 1.4 mL of the treatment solution which was added to a dental wick (Cremer S.A., Blumenau, Brazil). Concentrations in the range of 0.5 to 4 µL/mL were tested. For the control, the sugar syrup containing DMSO (10 µL/mL), and Tween 20 (5 µL/mL) was used. Five biological replicates were performed per treatment. Each replicate consisted of a glass vial (200 mL of capacity) containing 20 insects, totaling 100 insects per essential oil concentration and control treatment. The flasks were closed with foam and kept under laboratory conditions (25 ± 2 °C, 70 ± 5% relative humidity and a 12 h photophase). Mortality was evaluated after 24 h of exposure. The insects that did not walk or fly after receiving stimuli by a fine-tipped brush were considered dead.

### 4.3. Essential Oil Selectivity to Bees

The following bioassay was conducted based on the methodology outlined in the study by Britto et al. [56]. In this experiment, the foragers of *A. mellifera* and *P. helleri* were orally exposed to one concentration of *P. brevipedunculata* essential oil, referent to the LC_50_ estimated for *D. suzukii*. The *P. brevipedunculata* essential oil was prepared at a concentration of 2.4 µL/mL. To ensure proper dilution, the compound was mixed with a sucrose solution (50% [*m/v*]), Tween 20 (0.6 µL/mL), and DMSO (1.2 µL/mL). Regarding the establishment of appropriate control treatment, sugar syrup was prepared with the same concentrations of Tween 20 and DMSO. Both treatments were presented to the bees in 2 mL Eppendorf microtubes placed inside low-density plastic containers with a capacity of 500 mL. Each plastic container served as an experimental unit and contained ten bees, with five replicates per treatment (*n* = 50). To account for intercolonial variation, five different and healthy colonies were utilized. Aiming for a more consistent consumption during the experiment, the bees were fasted for one hour before being able to access the food. After five hours of exposure, all bees were provided with an uncontaminated diet (simple sucrose solution). Throughout the experiment, mortality and diet consumption were recorded at one, three, five, and 24 h from the beginning of the bioassay. The bees were housed in a biochemical oxygen demand (BOD) incubator, maintained at 33 ± 1 °C for *A. mellifera* and 28 ± 1 °C for *P. helleri*, both in the dark. A bee was considered dead if it exhibited no movement when gently prodded with a fine-hair brush.

### 4.4. Molecular Docking between Citral Isomers and D. suzukii and Pollinator Bees Targets

The sodium and chloride-dependent GABA receptors, AChE, and GST were selected as physiological targets. The amino acid sequences of the GABA receptors, AChE, and GST for predictions are available in the National Center for Biotechnology Information (NCBI) database with complete annotation. Thus, for GABA derived from *D. suzukii* we used the sequence XP_036671565.1 and for *A. mellifera* we used the sequence XP_395197.2. The Phyre2 server was used to build the 3D structure of proteins [57]. The quality parameter was evaluated by Prosa [58,59], including the constraints of the angles provided by the Ramachandran plot according to the Procheck tool [60,61]. A positive control was performed by simulating the interaction of GABA receptors from *D. suzukii* and *A. mellifera* with the GABA antagonist, the insecticide fipronil. For AChE, the amino acid sequences XP_036677504.1 and ANT80564.1 were downloaded for *D. suzukii* and *A. mellifera*, respectively, and a homology modeling approach was built using the Swiss Model Workspace https://swissmodel.expasy.org/ (accessed on 6 June 2021) with protein structure crashes and amino acid positioning at the active site [62], the Ramachandran plots [63], and QMEAN factor [64]. The PDB 6XYY template was found for acetylcholinesterase enzymes in Protein Databank with 99% identity for *D. suzukii* and 63% for *A. mellifera*. For GST, the amino acid sequences XP_036669239.1 and NP_001136128.1 for *D. suzukii* and *A. mellifera*, respectively, and the homology modeling approach was built using the Swiss Model Workspace. For the three receptors, the energy minimization was performed by Yasara forced field [65].

We prepared the citral isomers (α and β) using PubChem [66] at NCBI and stored them in SDF (Structure Data Format) for molecular docking predictions. The citral isomers and protein GABA receptor, AChE, and GST of both *D. suzukii* and *A. mellifera* were prepared with Autodock Tools 1.5.7 [67]. The best ligand–receptor complex which returned affinity energy values (kcal/mol) using the AutoDock Vina [68] was used to generate 2D interaction maps with Discovery Studio v21.1.0.20298 [69].

### 4.5. Statistical Analyzes

Concentration–mortality data were subjected to probit analysis using the SAS software 9.1 (SAS Institute, Cary, NC, USA). The mortality of the bees was compared using the *t*-test (*p* < 0.05) using the SigmaPlot Systat Software 14.0 (San Jose, CA, USA). The consumption of diets by bees was submitted to a repeated measures ANOVA to determine the effects of essential oil, bee species, and time using the SAS software 9.1.

## 5. Conclusions

In conclusion, our study highlights the efficacy of *P. brevipedunculata* essential oil as a promising tool to be incorporated in the integrated pest management (IPM) of *D. suzukii*, a relevant pest in pollinator-based crops. Thus, by demonstrating the minimal impact of *P. brevipedunculata* essential oil on the bees *A. mellifera* and *P. helleri*, our findings reinforce the safeness of such natural compounds for integrated pest management. However, despite the in silico predictions reinforcing the in vivo selectivity actions of the essential oil, further investigations (e.g., binding tests or electrophysiological recordings) are still required in order to clarify the potential modes of the actions of the essential oil or its constituents.

## Figures and Tables

**Table 1:** Chemical composition of the *Pectis brevipedunculata* essential oil.

Yield = 1.8%							
Constituents	RI_C_^1^	RI_L_^2^	%	Constituents	RI_C_^1^	RI_L_^2^	%
α-Thujene	928	924		Geraniol	1253	1249	3.4
α-Pinene	935	932	16.2	Carvenone	1261	1255	0.1
Sabinene	975	969	0.6	Geranial (=α-Citral)	1273	1264	33.9
β-Pinene	979	974	0.3	1-Tridecene	1295	1290	0.2
6-methyl-5-Hepten-2-one	988	986	1	2,4-Octanediol	1339	1339	0.2
Myrcene	992	988	0.3	trans-p-Menth-6-en-2,8-diol	1369	1371	0.1
Limonene	1032	1024	8.1	2-Undecen-1-ol	1374	1370	0.3
(E)-β-Ocimene	1050	1046	0.4	Geranyl acetate	1379	1379	0.2
3-methyl-1,2-Cyclohexanedione	1094	1089	0.3	β-Elemene	1396	1389	0.3
Linalool	1102	1095	1.2	(E)-Caryophyllene	1430	1424	0.4
exo-Isocitral	1147	1140	0.2	trans-Prenyl limonene	1467	1357	0.5
(Z)-Isocitral	1165	1160	1	Germacrene D	1492	1484	0.1
(E)-Isocitral	1183	1177	1.7	α-Alaskene	1520	1515	0.1
Terpinen-4-ol	1186	1180	0.2	δ-Cadinene	1526	1522	-
α-Terpineol	1200	1195	0.3	α-Muurolol (=Torreyol)	1649	1644	0.1
Nerol	1228	1227	1.1	Valerianol	1663	1657	-
Neral (=β-Citral)	1244	1235	26.7	Linoleic acid	2133	2132	-
Monoterpene hydrocarbons (%)							25.9
Oxygenated monoterpenes (%)							70.1
Sesquiterpene hydrocarbons (%)							1.4
Oxygenated sesquiterpenes (%)							0.1
Fatty acids and derivatives (%)							2
Total (%)							99.5

RI_C_^1^ = retention index calculated (DB-5ms column); RI_L_^2^ = retention index from the literature; bold = main constituents.

**Table 2:** Analysis of variance with repeated measures over time for the 24 h, including 5 h exposure to *Pectis brevipedunculata* essential oil-treated sugar syrup, with neotropical pollinator bees *Apis mellifera* and *Partamona helleri*.

Sources of Variation	df	*F*	*p*	
Between samples				
Essential Oil (EO)	1	3.86	0.067	
Species (S)	1	76.6	<0.0001 *	
EO × S	1	3.27	0.089	
Error	16	-	-	
	df_den_/df_num_	Wilks’ lambda	*F*	*p*
Within samples				
Time (T)	14/3	0.012	380.2	<0.0001 *
T × EOs	14/3	0.859	0.76	0.5327
T × S	14/3	0.071	61.0	<0.0001 *
T × EOs × S	14/3	0.890	0.57	0.6414

* Significant at *p* < 0.05.

## Data Availability

Data are contained within the article and Supplementary Materials.

## Supplementary Materials

The following supporting information can be downloaded at: https://www.mdpi.com/article/10.3390/plants13101392/s1, Figure S1: The Ramachandran plot of the protein models; Figure S2: Fipronil interacting with GABA receptors of *Drosophila suzukii* and *Apis mellifera*; Figure S3: Citral isomers interacting with AChE protein from *Drosophila suzukii* and *Apis mellifera*; Figure S4: Citral isomers interacting with GST protein from *Drosophila suzukii* and *Apis mellifera*; Table S1: The coordinates of the receptor grids on the targets.
