# Supplementary material for: Contributions of γ-Aminobutyric Acid (GABA) Receptors for the Activities of Pectis brevipedunculata Essential Oil against Drosophila suzukii and Pollinator Bees
Source: Plants (Basel). 2024 May 17;13(10):1392. doi: 10.3390/plants13101392 (PMC11124835; doi:10.3390/plants13101392)
Supplement: Supplementary file 1 [file plants-13-01392-s001.zip › plants-2969817-supplementary.pdf]

Supplementary Materials

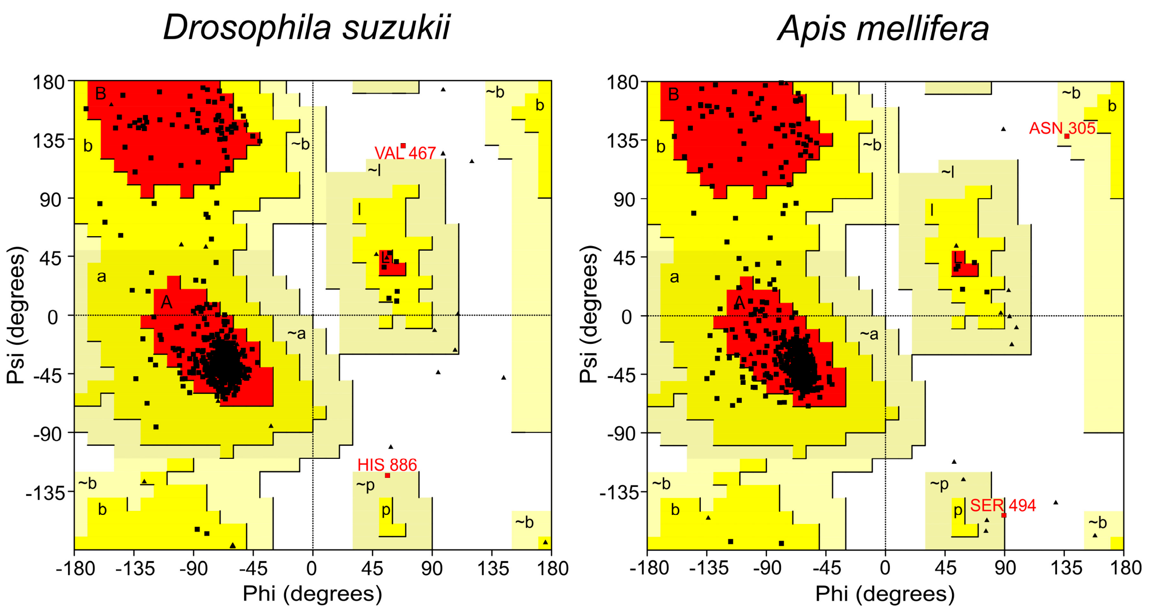

Figure S1. The Ramachandran plot of the GABA protein models. Based on an analysis of 118 structures of resolution of at least 2.0 Angstroms and R-factor no greater than 20%, a good quality model would be expected to have over 90% in the most favoured regions. Residues in most favoured regions [A,B,L]: *Drosophila suzukii* (425 – 92.2%), *Apis mellifera* (420 – 89.9 %); Residues in additional allowed regions [a,b,l,p]: *D. suzukii* (34 – 7.4 %), *A. mellifera* (45 – 9.6 %); Residues in generously allowed regions [~a,~b,~l,~p]: *D. suzukii* (1 – 0.2 %), *A. mellifera* (2 – 0.4 %); Residues in disallowed regions: *D. suzukii* (1 – 0.2 %), *A. mellifera* (0 – 0.0 %); Number of non-glycine and non-proline residues: *D. suzukii* (461 – 100.0 %), *A. mellifera* (467 – 100.0 %); Number of end-residues (excl. Gly and Pro): *D. suzukii* (5), *A. mellifera* (4); Number of glycine residues (shown as triangles): *D. suzukii* (40), *A. mellifera* (35); Number of proline residues: *D. suzukii* (24), *A. mellifera* (24); Total number of residues: *D. suzukii* (530), *A. mellifera* (530)

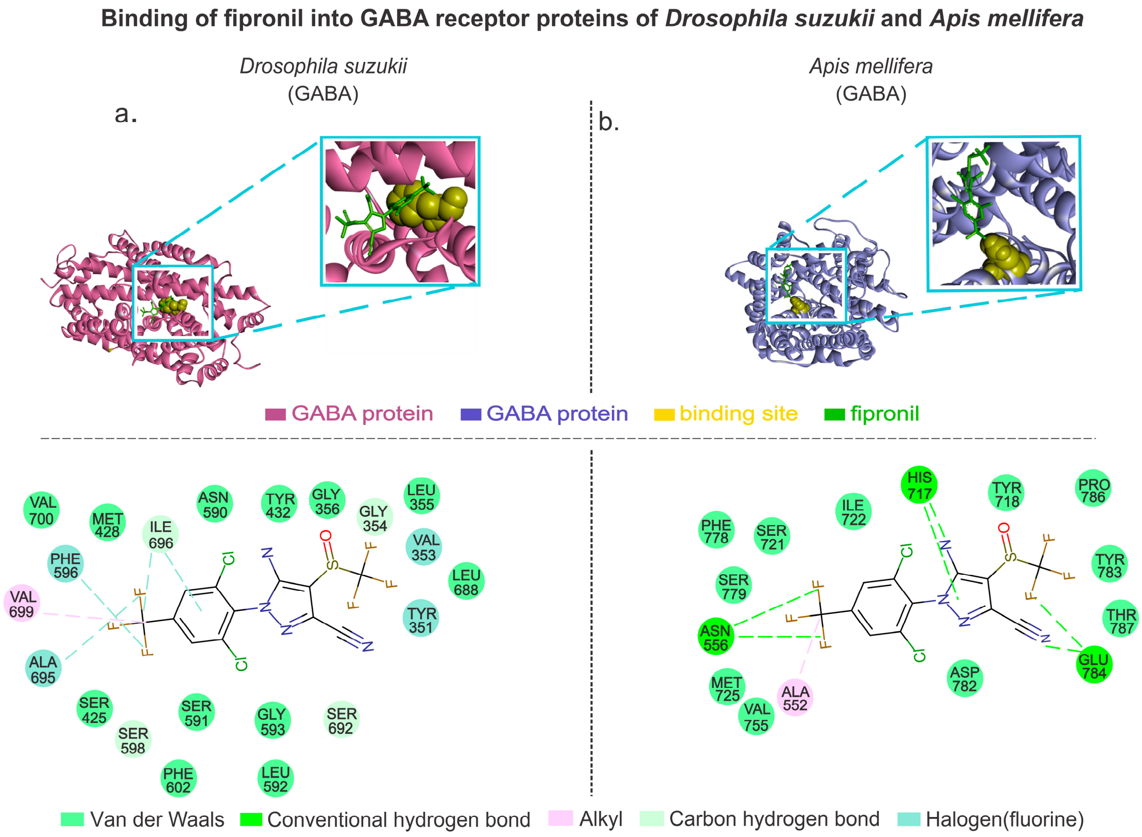

Figure S2. Fipronil interacting with GABA receptors of *Drosophila suzukii* and *Apis mellifera*. (a) Protein 3D structure of the *D. suzukii* GABA receptors with fipronil. b) Protein 3D structure of the *A. mellifera* GABA receptors with fipronil. c) 2D interaction maps showing the molecular interactions between amino acids from the binding site environments of *D. suzukii* GABA receptors with fipronil. (d) 2D interaction maps showing the molecular interactions between amino acids from the binding site environments of *A. mellifera* GABA receptors with fipronil.

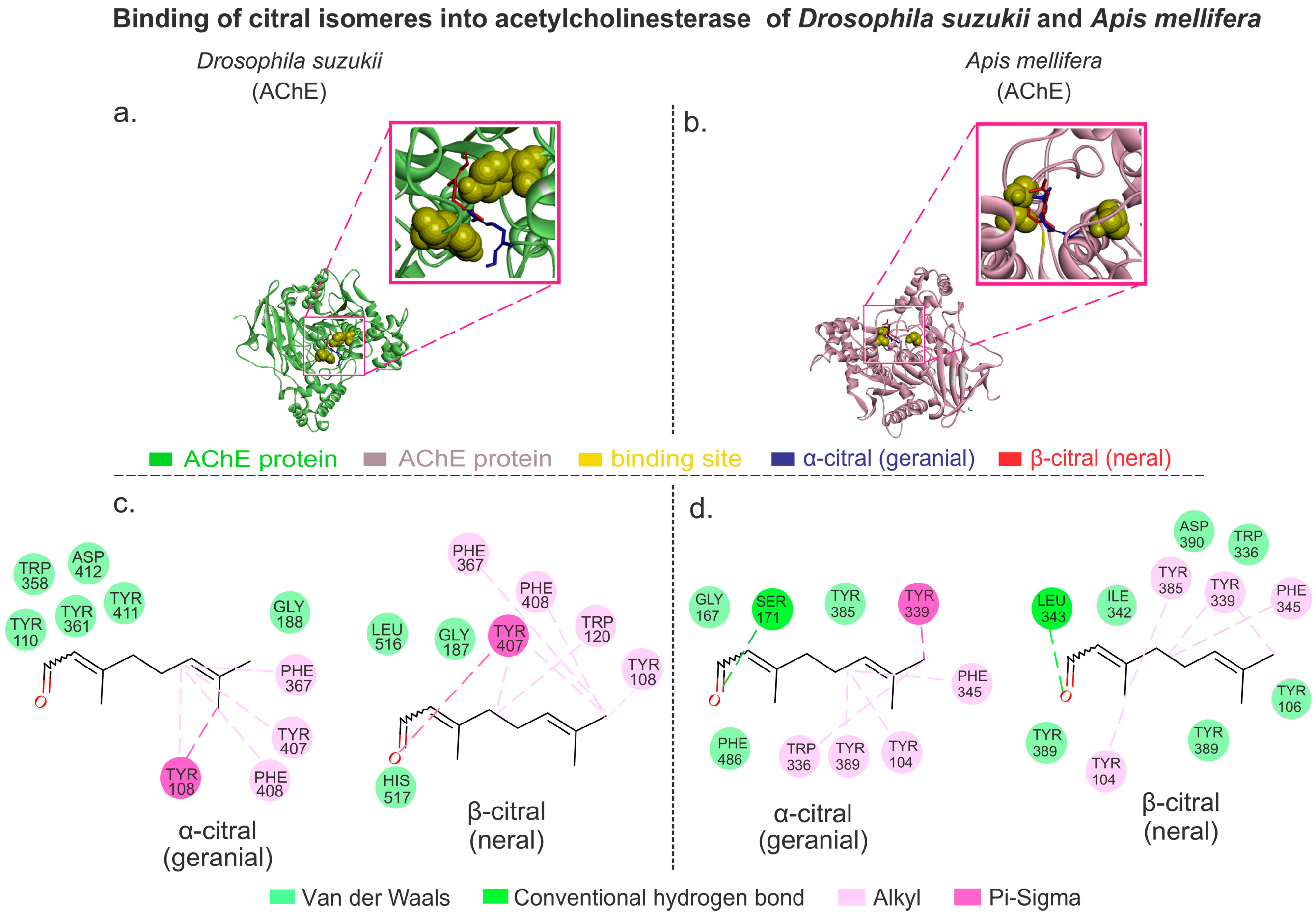

Figure S3. Citral isomers interacting with AChE protein from *Drosophila suzukii* and *Apis mellifera*. (a) Protein 3D structure of the *D. suzukii* AChE protein with the citral isomers. b) Protein 3D structure of the *A. mellifera* AChE protein with the citral isomers. c) 2D interaction maps showing the molecular interactions between amino acids from the binding site environments of *D. suzukii* AChE protein with the citral isomers. (d) 2D interaction maps showing the molecular interactions between amino acids from the binding site environments of *A. mellifera* AChE protein with the citral isomers

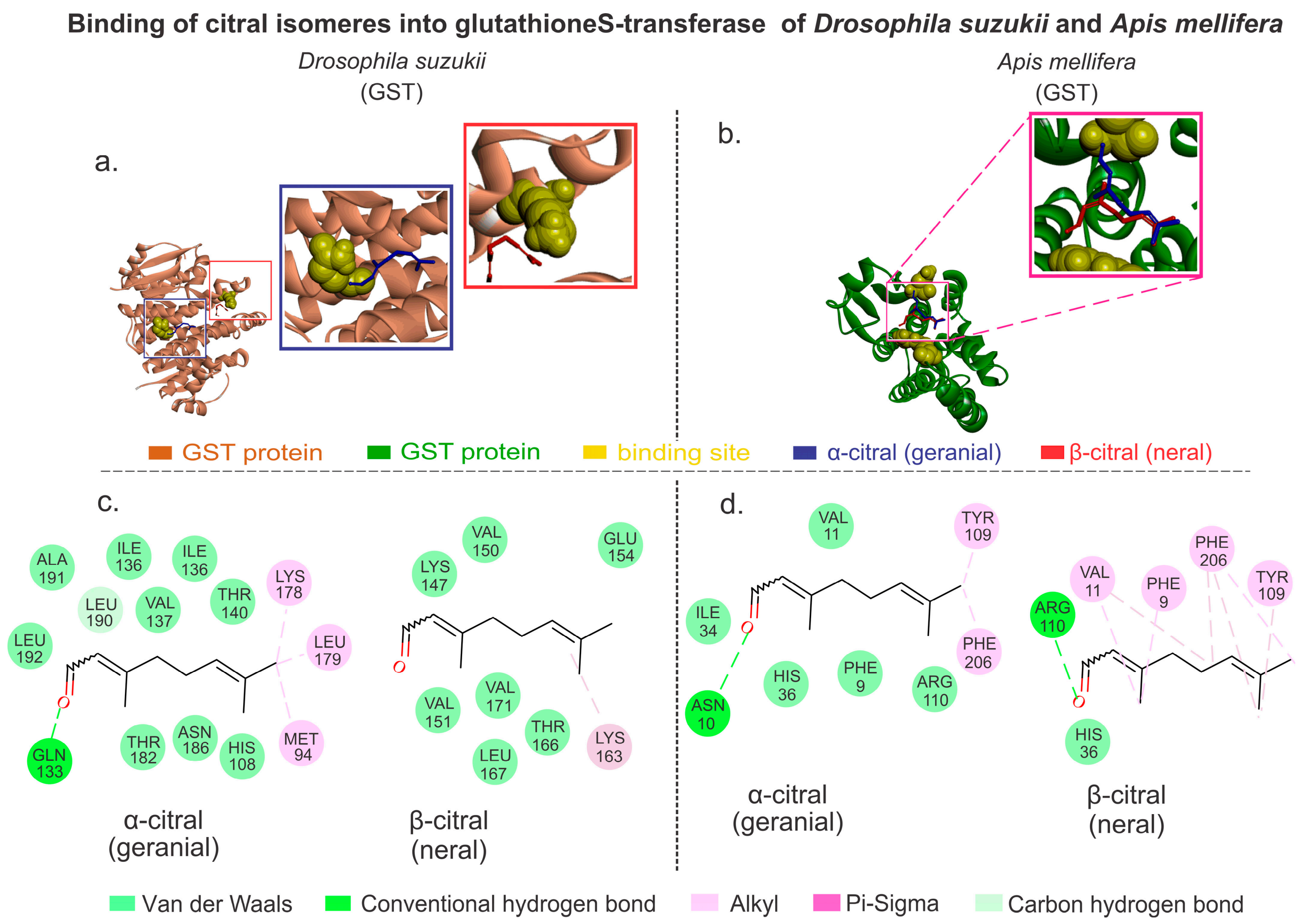

Figure S4. Citral isomers interacting with GST protein from *Drosophila suzukii* and *Apis mellifera*. (a) Protein 3D structure of the *D. suzukii* GST protein with the citral isomers. b) Protein 3D structure of the *A. mellifera* GST protein with the citral isomers. c) 2D interaction maps showing the molecular interactions between amino acids from the binding site environments of *D. suzukii* GST protein with the citral isomers. (d) 2D interaction maps showing the molecular interactions between amino acids from the binding site environments of *A. mellifera* GST protein with the citral isomers

Table S1 The coordinates of the receptor grids on the targets

**γ-aminobutyric acid (GABA) receptor**					
Drosophila suzukii			***Apis mellifera***		
α-citral	β-citral	Fipronil	α-citral	β-citral	Fipronil
124.039 6.693 42.925	124.039 6.693 42.925	124.039 6.693 42.925	-42.346 -0.589 54.926	-42.346 -0.589 54.926	-42.346 -0.589 54.926
**Acetylcholinesterase (AChE)**					
Drosophila suzukii			***Apis mellifera***		
α-citral	β-citral	Fipronil	α-citral	β-citral	Fipronil
24.426 63.184 9.919	24.426 63.184 9.919	*n.a*	24.326 63.259 10.076	24.326 63.259 10.076	*n.a*
**Glutathione-*S*-transferase (GST)**					
Drosophila suzukii			***Apis mellifera***		
α-citral	β-citral	Fipronil	α-citral	β-citral	Fipronil
56.191 20.534 6.667	56.191 20.534 6.667	*n.a*	-0.300 0.923 -0.368	-0.300 0.923 -0.368	*n.a*

*spacing 0.375; npts 40 40 40; *nt- not applicable*
